#  Comparison of Ebola virus infection routes and resulting disease in animal models

**DOI:** 10.1128/jvi.00081-26

**Published:** 2026-05-04

**Authors:** B. Ryder Gathright, Andrea Marzi

**Affiliations:** 1 Laboratory of Virology, Division of Intramural Research, National Institute of Allergy and Infectious Diseases, National Institutes of Health 828462 , , Hamilton, Montana, USA; Indiana University Bloomington, Bloomington, Indiana, USA

**Keywords:** filovirus, aerosol exposure, intranasal infection, intramuscular infection, mucosal infection, pathogenesis

## Abstract

Ebola virus disease (EVD) is a highly pathogenic and lethal disease caused by Ebola virus (EBOV). Endemic to Sub-Saharan Africa, EBOV has been causing global health concerns during infrequent EVD outbreaks since 1976, particularly during the 2013 –2016 epidemic in West Africa. Spread by contact of contaminated bodily fluids with mucous membranes or breaks in the skin, EBOV yields an incredibly high mortality rate and, as a result, has been designated a priority pathogen and a select agent to be studied exclusively in maximum containment laboratories. EBOV has historically been studied in animal models that accurately recapitulate disease progression, symptoms, and outcomes as seen in humans via more artificial infection routes such as intramuscular (IM) and intraperitoneal (IP) inoculation. There has recently been a concerted effort to characterize disease in animal models with mucosal inoculation routes with the goal to more accurately replicate human infection and development of EVD. This review aims to summarize and characterize the different inoculation routes used in the various animal models applied in EBOV research. We also outline the differences in disease progression between “artificial” and mucosal infection routes, comparing them to the course of EVD as seen in humans to highlight similarities and differences.

## INTRODUCTION

First discovered in 1976 in Zaire (now the Democratic Republic of the Congo [DRC]), Ebola virus (EBOV) is a filamentous virus in the family *Filoviridae* and the causative agent of Ebola virus disease (EVD). Symptoms of EVD include fever, rash, diarrhea, myalgia, coagulopathy and abnormal bleeding, and liver and spleen degeneration, leading to multisystem organ failure. EVD is highly lethal in humans, with mortality rates known to exceed 90% (1). Individuals succumb to EVD at an average of 9 days after onset of symptoms (2). The largest known outbreak to date occurred across West Africa from 2013 to 2016, resulting in over 28,000 cases and 11,000 deaths (1). Outbreaks happen infrequently, with six EVD outbreaks occurring in the DRC since 2020 (1).

Given the virulence as well as infrequency and remote location of EVD outbreaks, using human trials to determine the efficacy of medical countermeasures (MCMs) like therapeutics or vaccines is highly challenging. Animal models constitute the main instruments providing the opportunity to characterize pathogenesis and immune responses in a biological system rather than working with cell culture and *in silico* methods. The more closely an animal model recapitulates EVD as seen in humans, the more accurate the model will be for the evaluation of MCMs in preclinical efficacy studies. Furthermore, the U.S. Food and Drug Administration (FDA) considers the animal rule as a pathway for MCM approval for EVD and other diseases like it (3). However, different animal models present different challenges ranging from cost and availability to ease of handling and ethical concerns. Historically, animals have been inoculated with EBOV by artificial routes such as intramuscular (IM) and intraperitoneal (IP), allowing precise virus dosing. However, human-to-human EBOV transmission likely occur s through mucosal routes and percutaneous breaks in the skin, with various amounts of virus establishing a productive infection in a new host (4). Therefore, more recently, mucosal routes have been explored for the infection of animals with filoviruses, including intranasal (IN), oral, ocular , and aerosol (AE) (5–7). The changes in infection routes have uncovered differences in disease progression and pathogenesis and are summarized here.

## HUMANS

EBOV is transmitted through bodily fluids like blood from an infected person. When EBOV enters the body via mucous membranes or breaks in the skin, it primarily targets dendritic cells and macrophages at the site of infection. These infected cells subsequently transport the virus to the draining lymph node and then on to the liver and spleen (8). After at least 3 days of incubation period, early signs of EVD begin relatively nonspecifically with myalgia, fatigue, malaise , and fever 8–10 days post-exposure (9, 10). The next stage of EVD several days later (10–14 days post-exposure) includes abdominal pain, vomiting , and diarrhea, all containing high titers of virus (11, 12). A characteristic petechial rash, accompanied by coagulopathy and bleeding, can but does not always occur during this stage and is often observed in the gums, eyes, etc. (11, 12). Cytokine dysregulation is commonly observed in human cases, with soaring interleukin (IL) and tumor necrosis factor (TNF) levels and impaired interferon (IFN) responses resulting in a cytokine storm in the more severe cases, leading to increased likelihood of lethality (12, 13). Leukopenia, thrombocytopenia , and neutrophilia are commonly observed in EVD patients (12, 14, 15). As the disease progresses, organ damage caused by viral replication, vascular leakage , and cytokine storm becomes more evident , and terminal cases of EVD will present with systemic infection resulting in kidney damage, rhabdomyolysis of the muscles, and encephalitis, all culminating in multiorgan failure and death (12). However, human EVD is not uniformly lethal , with survivors beginning to improve 6 –10 days after disease onset , as evidenced by the ~ 50 % survival rate in the 2013 –2016 West African EVD outbreak (12).

## NONHUMAN PRIMATES (NHPs)

NHPs are regarded as the gold standard in EBOV research, particularly rhesus and cynomolgus macaques. Cynomolgus macaques have a compressed disease course of EVD compared to rhesus macaques, presenting a more stringent model (16). Therefore, cynomolgus macaques are historically used for vaccine efficacy studies , while rhesus macaques are preferably used to evaluate therapeutics as they provide a prolonged treatment window (16). Both macaque species closely recapitulate EVD as seen in human patients when infected with wild-type (WT) EBOV through numerous routes (17). Historically, macaques are infected with WT-EBOV IM in the caudal thigh, allowing for controlled deposition of the inoculum and mimicking accidental needle stick exposures to humans. IM is an artificial exposure route presenting a high bar for stringent MCM development, however, it does not accurately recapitulate natural exposure, which occurs via mucosal surfaces and may result in slower disease progression (4).

When infected with 1,000 PFU IM, rhesus macaques quickly develop fever (3 days post-infection [DPI]) followed by petechial rash at 5–6 DPI (8, 17). Signs of disease include high-titer viremia first appearing within 3 DPI, evidence of coagulation abnormalities, thrombocytopenia, a drop in hematocrit and hemoglobin, and, in rarer cases, bleeding from orifices and mucous membranes (17, 18). In addition, immune system dysregulation is observed with lymphopenia and a cytokine storm (Table 1) along with accumulation of IFNs, ILs , and macrophage inflammatory protein (MIP ) (17, 19, 20). The disease is uniformly lethal with an average time to death of 6.5 DPI; at necropsy, an enlarged and friable liver, enlarged spleen, and lymph nodes with hemorrhage and edema have been commonly described (17, 21). Human EVD following a needle stick exposure follows roughly the same disease progression timeline as observed in NHPs IM infected with 1,000 PFU, which is much faster compared to human EVD after natural exposure, which results in a protracted disease course. While IM is the standard route of infection, NHPs are also susceptible to exposure and develop lethal disease after IP inoculation, although it is not a commonly used route (22). When infected IP with 6 PFU of WT-EBOV, naïve cynomolgus macaques developed disease, and all succumbed between 5 and 7 DPI, exhibiting the same signs of disease described above for rhesus macaques (22).

**TABLE 1:** Disease signs and outcomes of NHPs infected with WT-EBOV^*a*^

Disease sign	IP	IM	IN	Oral	Ocular	Aerosol
Fever	+	+	+	+	+	+
Weight loss	+	+	+	+	+	+
Viremia	+	+	+	+	+	+
Petechial rash	+	+	+	+	+	+
Hemorrhages	+	+	+	+	+	+
Anemia	+	+	+	+	+	+
Coagulopathy	+	+	+	+	+	+
Lymphopenia	+	+	+	+	+	+
Thrombocytopenia	+	+	+	+	+	+
Liver damage	+	+	+	+	+	+
Cytokine storm	+	+	n.d.	n.d.	+	+
Lethality	Uniform	Uniform	Uniform (more prolonged than IM/IP)	Dose dependent (10,000 PFU—60 % mortality)	Dose dependent (100 PFU—60 % mortality; 10,000 PFU—100 % lethality)	Dose dependent (1,000 PFU—uniform lethality)

^*a*^ IP, intraperitoneal; IM, intramuscular; IN, intranasal; +, present; n.d., no data.

NHPs develop fatal EVD after mucosal infection, including the IN route (Table 1). A study compared disease progression and pathogenesis in cynomolgus macaques after IN (two groups: 120 and 500 PFU) and IM (100 PFU) WT-EBOV infection and found notable differences (5). Time to death was longer in the IN group compared to the IM group, with an average of 8.9 DPI compared to 7 DPI, respectively. Clinical signs of disease took longer to appear in IN-infected NHPs. The elongation of the disease course after mucosal infection is consistent in both cynomolgus and rhesus macaques (23, 24) and aligns with that observed in human infections (12). While most clinical parameters were similar between the IN and IM groups, there was increased liver damage documented in the IM group (5). Viremia was detected in the IN groups as early as 3 DPI; in contrast, the IM group presented with viremia at 5 DPI. The differences in viremia and liver damage suggest differences in early replication sites between these groups (5). In addition, pronounced hemorrhaging of the gastrointestinal (GI) tract and coagulation abnormalities were found in the IN group. Platelet count started to drop in both IN and IM groups around 3 DPI (5). Other mucosal routes of infection include the conjunctival and oral routes; however, higher viral infection doses compared to IM and IN are required in order to cause fatal disease (25). Survival rates are highly dose-dependent for conjunctival infection, causing uniform lethality at 10,000 PFU and 80 % survival at 100 PFU WT-EBOV infection (26). In contrast, orally infected NHPs develop disease after 10,000 PFU WT-EBOV infection, but 40 % of these infected NHPs survived (7). In a different study, oral infection with WT-EBOV resulted in lethality in NHPs with doses as small as 100 PFU (10 PFU results in no clinical illness) (27), highlighting that other parameters like NHP species and EBOV isolate contribute to the disease outcome. EBOV infection through the aerosol route can also be achieved in NHPs. Infection by this route is similarly dose-dependent; however, high doses of EBOV cause uniform lethality (28, 29). For example, when infected with 1,000 PFU AE, uniform lethality is achieved 7 –10 DPI (29). As previously mentioned, differences in disease progression in macaque species have been documented; however, for each species, disease signs and progression are consistent when infected via the aerosol route (28). The relevance of aerosol infection was highlighted in a transmission study when rhesus macaques infected IM with EBOV transmitted EBOV to two of three naïve macaques without direct contact (3 meters of distance in the same room) (30). Pathological analysis revealed evidence of mucosal transmission and lung pathogenesis. Given the significance of natural mucosal infection and transmission in NHPs as well as humans, studies recapitulating EVD using mucosal infection routes are important to better our understanding of disease progression and the identification of novel treatment options.

NHPs, particularly macaques, are rightfully considered to be the “gold standard” in filovirus research as they can be productively infected with WT-EBOV by various routes and accurately recapitulate EVD as seen in humans (16). They remain an integral part of countermeasure development and approval by the FDA’s animal rule despite the challenges surrounding availability, acquisition cost, and difficulty to house and handle (3).

## MICE

While mice do not recapitulate human EVD to the extent of NHPs, the comparatively low cost, ease of handling, and abundance of knock-out strains and reagents available for analysis have led to mice being the most used animal species in the study of filovirus pathogenesis (31). In general, wild-type mice inoculated with WT-EBOV do not develop disease regardless of the inoculation route (32). In order to acquire virulence and cause disease in WT mice, mouse-adapted (MA-) EBOV was generated through serial passaging in suckling mice (32). When inoculated with MA-EBOV IP, disease and uniform lethality were achieved in WT mice (Table 2). When administered subcutaneously (SC) or IM, MA-EBOV does not cause disease in WT mice and rather confers protection against subsequent IP reinfection (33). When infected IP, mice display some characteristics of EVD as seen in humans and NHPs, including viral damage to the liver and spleen, resulting in elevated hepatocellular enzymes and degeneration/necrosis, thrombocytopenia, and occasional/mild coagulopathy (14, 31). High levels of EBOV replication occur in the spleen, liver, and lymph nodes, resulting in organ degeneration, lesion formation, and ultimately necrosis (31). Viremia is detected early, alongside pro-inflammatory cytokines like TNF-α and MCP-1. This heightened cytokine response becomes dysregulated in cases of human EVD and in various animal models of EVD, resulting in lethality (12, 14, 31). The observed weak IFN-α and strong TNF-α responses have been shown to be a result of a downregulation of IFN signaling (34). Notably, during SC infection, an early and more prominent IFN-α response is observed, resulting in a greater innate immune response conferring protection (34). Curiously, mice infected with MA-EBOV do not develop a petechial rash, a common sign of human and NHP EVD, and show inconsistent coagulopathy (31).

**TABLE 2:** Disease signs and outcomes of mice infected with MA-EBOV or WT-EBOV^*a*^

Mice	Virus	IP	IM	SC	IN	Oral/ oro-nasal	Ocular
Laboratory strains, including Balb/c and ICR (CD-1)	MA-EBOV	Viremia, spleen/liver/lymph node degeneration, weak interferon response, and strong TNF response; uniform lethality	No disease	No disease	n/a	n/a	n/a
	WT-EBOV	No disease	No disease	No disease	n/a	n/a	n/a
IFNAR ^ −/ − ^ mice	MA-EBOV	Viremia and liver/spleen degeneration; uniform lethality	n/a	Viremia and liver/spleen degeneration; uniform lethality	n/a	n/a	n/a
	WT-EBOV	Viremia and liver/spleen degeneration; uniform lethality	n/a	Viremia and liver/spleen degeneration; uniform lethality	n/a	n/a	n/a
NSG-huPBL (humanized mice)	MA-EBOV	Spleen and liver degeneration; human lymphocyte apoptosis; uniform lethality	n/a	n/a	Viremia, high viral load in liver with degeneration, and pro-inflammatory cytokines	n/a	n/a
	WT-EBOV	Uniform survival	n/a	n/a	n/a	n/a	n/a
Hu-NSG-A2	WT-EBOV	Spleen and liver degeneration; 75%–100% lethality dependent on the level of engraftment	n/a	n/a	Viremia, high viral load in liver with degeneration, and pro-inflammatory cytokines; ~92% lethality	n/a	n/a
Hu-NSG-SGM3	WT-EBOV	Spleen and liver degeneration; 0%–50% lethality (dose dependent)	T cell activity in the liver and spleen; 66% lethality	n/a	n/a	n/a	n/a
NSG-huBLT	WT-EBOV	Hepatic damage and upregulation of human chemokines and cytokines; 50%–100% lethality	n/a	n/a	n/a	n/a	n/a

^*a*^ IP, intraperitoneal; IM, intramuscular; SC, subcutaneous; IN, intranasal; n/a, not applicable.

WT-EBOV infection may result in EVD-like disease in mice if the mice are immunodeficient or possess (partly) humanized immune systems (31). Mice lacking the IFN-α/β receptor (IFNAR^ −/−^ ) have been shown to succumb to WT-EBOV infection within one week (35). Fatal disease is uniformly observed in IFNAR ^−/−^ mice after infection with MA-EBOV or WT-EBOV via the IP and SC routes (Table 2), with the IP route resulting in shorter time to death (35). The same can be observed in STAT1 ^−/−^ mice and immun ocompetent WT mice treated with anti-IFN-α/β antibodies (35). Large amounts of replicating virus are found in the spleen and liver of immunodeficient mice infected with WT-EBOV (35). Observations from infected IFNAR ^−/−^ and STAT1 ^−/−^ mice provide evidence that protection against lethal EBOV infection is due, at least in part, to a functional type I IFN response (35). However, due to the immunodeficient nature of these mice, it is difficult to glean any immunological findings that translate to human EVD (31).

Mice engrafted with human immune cells, humanized mice, have been shown to develop EVD-like disease after WT-EBOV infection (31). There are several different kinds of mice with humanized immune systems (HIS). NSG-huPBL mice are engrafted with peripheral blood lymphocytes and do not succumb when infected IP with 1,000 plaque-forming units (PFU) WT-EBOV; however, they succumb uniformly to disease after infection with the same dose and route of MA-EBOV (36). Hu-NSG-A2 mice are engrafted with hematopoietic stem cells (HSCs) and have functional HLA-A2-restricted T cell responses (36). IP infection of Hu-NSG-A2 mice with WT-EBOV resulted in near uniform lethality (Table 2), with the likelihood of lethality increasing with increasing levels of engraftment (% human CD45+ of total peripheral blood leukocytes) (36). Signs of disease included elevated liver enzyme levels and liver hemorrhaging along with an enlarged spleen (36). Other types of HIS mice infected with WT-EBOV develop lethal disease by administration through the IM and IN routes in addition to the IP route (36). Hu-NSG-SGM3 mice express human IL-3, GM-CSF, and SCF cytokines. IM infection with as little as 10 PFU WT-EBOV resulted in 66 % lethality (36). A group of mice infected with 1,000 PFU WT-EBOV IP showed 50% lethality (Table 2); in contrast, 10 PFU resulted in uniform survival (36). In another study, Hu-NSG-A2 mice were infected IN with WT-EBOV to replicate mucosal spread of EBOV (37). When infected with 1,000 FFU of WT-EBOV, the IN route resulted in > 92% lethality, with mice succumbing consistently 10–20 days post-infection (DPI). High levels of viremia and aspartate aminotransferase (AST) were identified in the serum of those that succumbed (37). Yet another study reported that humanized mice infected IN with WT-EBOV do not show differences in time to death compared to IM-infected humanized mice (38).

Mice are a great starting model for pathogenesis and initial MCM efficacy studies; there is an abundance of tools available to analyze immune responses to infection and the MCM. In addition, knockout mice can be easily generated, highlighting the usefulness of this animal species in EVD research.

## HAMSTERS

Much like mice, Syrian golden hamsters are readily available as laboratory animals for study. They also do not develop EVD when infected with WT-EBOV. However, MA-EBOV infection results in lethal disease when EBOV is administered IP (25, 39). Like mice, hamsters infected SC with MA-EBOV do not develop disease. Signs of EVD-like disease in hamsters include cytokine dysregulation in the form of type 1 interferon suppression, lymphocyte apoptosis and degradation, and necrosis of the spleen, liver, and lymph nodes (Table 3), all similar to disease presentation in humans and NHPs (25, 31, 39). Unlike mice, hamsters exhibit coagulopathy similarly to NHPs and humans (25, 39). However, hamsters do not display a petechial rash, a dissimilarity from human EVD (14, 39).

**TABLE 3:** Disease signs and outcomes of Syrian golden hamsters, Hartley guinea pigs, and ferrets infected with MA-EBOV, GPA-EBOV, and WT-EBOV^*a*^

Animal species	Virus	Disease signs	IP	IM	SC	IN	Oral or oronasal	Ocular
Syrian golden hamsters	MA-EBOV	Fever		n/a	No disease	n/a	n/a	n/a
		Weight loss						
		Viremia	+					
		Petechial rash						
		Hemorrhages						
		Anemia						
		Coagulopathy	+					
		Lymphopenia						
		Thrombocytopenia	+					
		Hepatic damage	+					
		Lymph degeneration						
		Cytokine storm						
Guinea pig (Hartley)	GPA-EBOV	Fever	+	n/a	+	+	n/a	n/a
		Weight loss	+		+	+		
		Viremia	+		+	+		
		Petechial rash						
		Hemorrhages						
		Anemia						
		Coagulopathy	+		+			
		Lymphopenia	+		+			
		Thrombocytopenia	+		+			
		Hepatic damage	+		+			
		Lymph degeneration						
		Cytokine storm						
Ferrets	WT-EBOV	Fever	n/a	+	n/a	+	+	Subclinical infection
		Weight loss						
		Viremia		+		+	+	
		Petechial rash		+		+	+	
		Hemorrhages						
		Anemia						
		Coagulopathy		+		+	+	
		Lymphopenia		+		+	+	
		Thrombocytopenia						
		Hepatic damage		+		+	+	
		Lymph degeneration						
		Cytokine storm		+		+	+	

^*a*^ IP, intraperitoneal; IM, intramuscular; SC, subcutaneaous; IN, intranasal; +, present; n/a, not applicable.

Hamsters were not widely used in infectious disease research prior to the COVID-19 pandemic as tools to perform in-depth immune response analysis were lacking. However, since the COVID-19 pandemic, reagent development has intensified with a variety of tools available to decipher T cell and cytokine responses.

## GUINEA PIGS 

Much like mice and hamsters, guinea pigs do not develop disease after WT-EBOV inoculation. Guinea pig-adapted EBOV (GPA-EBOV) is generated by serial passaging of WT-EBOV in guinea pigs and, when administered IP to guinea pigs, the animals developed EVD-like disease (40, 41). Unlike mice and hamsters, guinea pigs recapitulate the hallmark symptoms of human EVD more accurately (42). This not only provides a higher predictive value for efficacy testing of MCMs in NHPs and humans but also allows for a more accurate understanding of disease development depending on inoculation routes. Guinea pigs infected IP with GPA-EBOV develop uniformly lethal disease and succumb at 8–9 DPI (41, 42). IP infection yields many of the hallmark symptoms of EVD, including weight loss, thrombocytopenia, increase in lymphocyte count and liver enzyme levels, and viremia (42). GPA-EBOV initially replicates to high titers in the liver and spleen before spreading to the kidneys, lungs, pancreas, and adrenal gland (41). In contrast to mice, guinea pigs develop a fever, show coagulation abnormalities consistently, and have a generally slower disease progression (42). Guinea pigs have also been shown to be susceptible to SC infection with GPA-EBOV, which results in delayed disease onset as well as lower viremia (42). Despite the delayed onset of disease, SC infection is uniformly lethal , and the signs of disease are similar to those observed in IP-infected guinea pigs (42).

**Fig 1:**
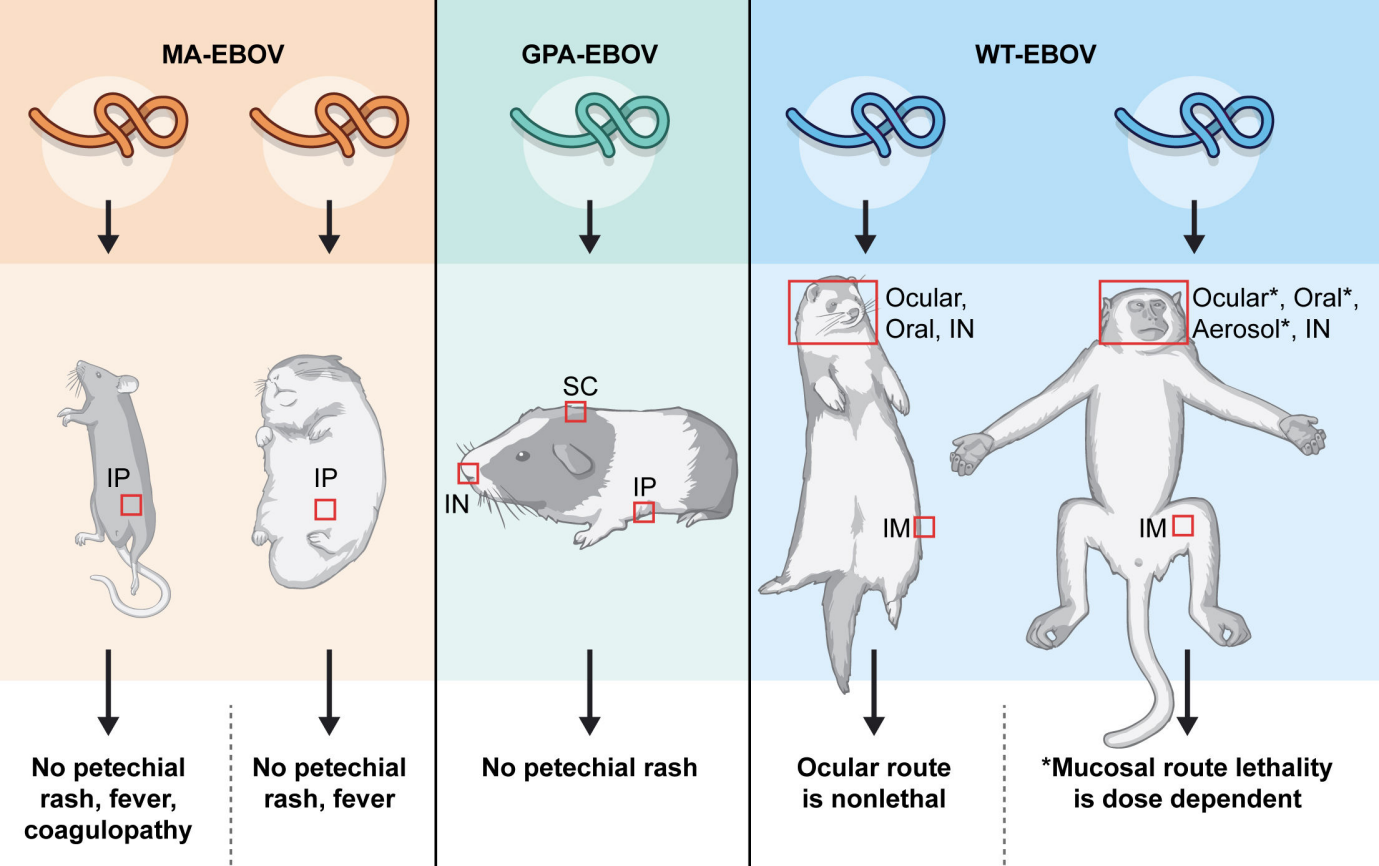
EBOV inoculation routes and disease outcome in animal models. Mice and hamsters intraperitoneally (IP) infected with mouse-adapted Ebola virus (MA-EBOV) recapitulate some signs of human Ebola virus disease (EVD), except no petechial rash, fever, or coagulopathy. Guinea pigs can be productively infected with guinea pig-adapted (GPA) EBOV via the IP route as well as subcutaneous (SC) and intranasal (IN) routes, mainly missing the development of a petechial rash. Ferrets and non-human primates (NHPs) can be productively infected with wild-type (WT) EBOV via several routes, particularly many different mucosal routes. Notably, ocular exposure in ferrets is nonlethal; lethality after mucosal exposure in NHPs is highly dose- dependent.

Notably, guinea pigs are also susceptible to mucosal infection via the IN route (Fig. 1; Table 3). Uniform lethality is observed in guinea pigs infected IN with 10,000 LD_50_ GPA-EBOV, with an average time to death of 8.7 DPI (longer than the 7.3 DPI observed in the IP group infected with the same dose) (43). Signs of disease include rapid weight loss, fever, hypothermia, and early viral shedding, particularly present in nasal swabs (43). Due to the efficient viral shedding, the virus can be more readily transmitted to naïve guinea pigs when compared to IP-infected donor guinea pigs (43). IN infection of guinea pigs, while causing similar pulmonary signs of disease as the IP group, also resulted in significant pneumonia and more severe lesions (43). The alveolar air space in the lungs of these guinea pigs contained large amounts of degenerating macrophages and granulocytes and necrotic debris, as observed by histologic analysis (43).

Guinea pigs have a higher predictive value for MCM development for EBOV compared to mice. However, there are only a limited number of reagents available to analyze cellular immune responses and cytokine responses, hampering the use of this model.

## FERRETS

Ferrets belong to the carnivorous family *Mustelidae* and differ from classical rodent disease models. In the last decade, ferrets have been used more frequently to study EBOV pathogenesis as they do not require virus adaptation. Instead, ferrets develop lethal disease after WT-EBOV infection (44). Ferrets have several advantages compared to other nonrodent animal models like NHPs; they are more widely available and more cost-effective to obtain. Enabling larger group sizes to be studied and easier handl ing in the BSL4 laboratory. Ferrets infected with EBOV by the IM or IN routes develop uniformly lethal disease at 5–7 DPI with doses as low as 0.1 PFU (44). Signs of disease include weight loss, fever, coagulopathy and thrombocytopenia, viremia, and immune dysregulation, as evidenced by lymphopenia, neutrophilia, and increased levels of TNF-α as well as numerous other pro-inflammatory cytokines and chemokines (44). High viral titers are found in the liver, spleen, kidneys, and lungs at the time of death regardless of the infection route (44). Infected ferrets also demonstrate petechial rash, a hallmark of EVD in humans and NHPs, which is not consistently observed in the rodent models (44). Much like guinea pigs, ferrets infected IN present with severe lung pathology including pneumonia, bronchiolitis, and perivasculitis (44).

Ferrets are sensitive to infection by mucosal routes including oral, ocular, and oronasal (6, 45). Ferrets ocularly infected with up to 100 PFU of WT-EBOV develop minimal to no disease and survived; pathologic analysis showed evidence of subclinical infection (6). In contrast, oral and oronasal infection of ferrets resulted in uniformly lethal disease. Most ferrets succumbed 5–6 DPI, though one ferret infected with 1 PFU WT-EBOV via the oral route did not succumb until 10 DPI (6). Signs of disease for these routes include fever, petechial rash, high viral titers in liver, spleen, and kidneys, coagulopathy, lymphocytopenia, and neutrophilia (Table 3). These signs are consistent with those of other infection routes, both mucosal (IN) and more artificial (IM) (6). Aerosolized WT-EBOV can also be used to infect ferrets. A study comparing IM and AE infection in ferrets found that virus in the spleen and liver as well as viremia appeared earlier in the AE model (46). Inversely, fever, clinical signs of disease, systemic inflammation, and liver pathology occurred earlier in the IM group. Furthermore, the AE group took longer to succumb to disease (6–7 DPI rather than 5–6 DPI for IM) (46). Lung involvement was observed in the AE group only, while the IM group displayed renal damage.

Uniform lethality is achieved in ferrets with WT-EBOV infection by multiple routes, notably mucosal routes, in addition to the more artificial IM route. Time to death is relatively uniform regardless of the infection route, though it has been noted as slightly longer for certain mucosal routes. While small rodents are more readily available and more cost-effective, ferrets develop disease after WT-EBOV infection and recapitulate EVD more robustly and consistently. However, at least compared to mice, the analysis of immune responses is hampered by the lack of available reagents thus far, impacting the use of this model for EBOV MCM development.

## DISCUSSION

When modeling and studying EVD in animals, the impact of the infection route is significant on the observed disease progression (Fig. 1). In general, EBOV IP injection is most commonly used in rodents, with the virus infecting macrophages in the abdominal cavity first before reaching the spleen via the lymphatic system (47). This artificial infection route, while enabling control over the infection dose and volume, results in rapid disease development and is often lethal, which is ideal for MCM development but is not recapitulating a natural disease course. In contrast, mucosal infection routes target macrophages and dendritic cells in the upper respiratory tract. These cells subsequently migrate to regional lymph nodes before reaching the spleen and liver (12). The infection routes determine pathologic differences as discussed above, including damage to the lungs and the gastrointestinal tract. Differences in liver pathology have also been noted, with IM injection of EBOV resulting in higher levels of hepatic damage and degeneration. The difference in artificial (IP and IM) vs mucosal/natural infection route (mucous membrane and percutaneous) also affects time to death, onset of illness, and levels of viremia (5–7, 28, 29, 43, 46). While the artificial route exposures result in a consistent yet contracted disease course and present a high bar for MCM development, they do not faithfully recapitulate human disease and, therefore, caution should be used when interpreting preclinical data. In general, symptom onset occurs earlier in animal models including NHPs; however, the extensive dehydration observed during human EVD caused by vomiting and diarrhea (12) is often not present in animal models.

When exposed to EBOV by mucosal routes, NHPs, guinea pigs, and ferrets display delayed signs of EVD and a longer disease course compared to animals infected via artificial routes such as IM or IP. IN infection in guinea pigs and NHPs both resulted in uniformly elongated disease courses. While most infection routes for ferrets resulted in similar time to death, the oral and aerosol routes resulted in longer disease courses. These longer disease courses more accurately represent EVD, as seen in humans, allowing more in-depth characterization of EVD and more accurate testing and use of MCMs in animal models that are infected via mucosal routes. Longer disease courses and surviving EBOV infection by mucosal routes would also enable us to expand our understanding of sequelae and viral recrudescence, as observed in human EVD survivors.

Severe pathology in the GI tract and lung is associated with mucosal infection routes in animal models. GI involvement is a common symptom of EVD in humans (5). Pulmonary symptoms have likewise been reported in EVD patients, becoming more pronounced as the disease course progresses (48). By more accurately recapitulating signs of human EVD in animal models, researchers have gained a better understanding of the effect of MCMs on specific aspects of the disease. Similarly, mucosal infection enables the investigation of virus transmission between animals at much higher rates compared to other routes. Early rapid shedding of the virus resulted in increased rates of transmission from IN-infected guinea pigs to naïve cage mates, resulting in much higher mortality rates in naïve animals specifically when spread from IN-infected guinea pigs. NHPs challenged via the mucosal route transmitted EBOV to naïve NHPs when not in direct contact, with lung pathology indicating mucosal transmission. Mucosal challenge has been shown to recapitulate the time frame, pathology, and symptoms of human EVD more faithfully. Using mucosal challenge routes in animal models may better mimic human transmission and EVD, providing more realistic models of EVD that we can study and treat. While mucosal infection routes may better recapitulate EVD, there are obstacles in using these methods. One of the benefits of using artificial routes is uniform lethality. Animal models showing uniform lethality are sought after for MCM development because statistical significance can be achieved with fewer animals (49). Mortality rates of NHPs infected via mucosal routes are highly variable, dose-dependent, and would therefore require greater numbers of animals or lead to uncertainty in the cause of survival in the case of MCM studies. Furthermore, artificial routes create a more condensed and relatively uniform time to death, minimizing the length of experiments and reagents needed. As previously discussed, animal studies using mucosal routes can vary significantly in time to death. Gaps in understanding how the infection route impacts the course of EVD remain. Further work must be done to fully characterize variation in the immune response, viral replication, outcomes, etc. One of the newer focuses of EBOV research involves sequelae and recrudescence in survivors of EVD. Sequelae being the conditions resulting from a prior disease not necessarily linked to viral persistence and recrudescence being the recurrence of EVD symptoms along with potential reinfection are distinct conditions following initial infection and EVD (50). After the West African epidemic, it became clear that many survivors experience sequelae, with cases occasionally becoming severe and even life-threatening (51). In terms of persistence, viral RNA has been found among EVD survivors in certain parts of the body such as parts of the eye and semen, where the immunological environment has allowed for its survival, causing subsequent outbreaks through contact, such as sexual transmission (51). Using mucosal routes in animals to model the EVD course more accurately could allow researchers to replicate cases of sequelae and recrudescence in the lab setting and better understand its causes and ways to treat it.
